# The first report of Tocilizumab for Darier disease with summer exacerbations^☆^

**DOI:** 10.1016/j.abd.2022.10.016

**Published:** 2024-03-04

**Authors:** Lele Chen, Wenwen Wang, Sen Zhou, Zhiming Li

**Affiliations:** The First Affiliated Hospital of Wenzhou Medical University, Wenzhou, Zhejiang, China

Dear Editor,

Darier Disease (DD), also known as keratosis follicularis, is a rare dermatosis caused by mutations in the ATP2A2 gene. There are no currently validated curative treatments available, besides oral retinoids, and most cases are treated symptomatically.^1^ Here, we reported the first case of using a monoclonal antibody against human IL-6R (Tocilizumab) in the treatment of summer exacerbations of DD.

A 23-year-old woman presented with a more than 10-year history of multiple pruritic yellow–brown keratotic papules covered with greasy scales. Physical examination showed diffuse red, skin-colored corn-like papules with keratosis, confluence in a flaky distribution, and multiple pustules and crusts (Fig. 1A). During the summer or before menstruation, her symptoms became severe and were characterized by more papules and pustules visible on her forearm with a burning sensation and intense itching that made it impossible to sleep. The patient had no relevant medical or family background. The results of laboratory tests showed markedly increased levels of serum IL-6 24.40 pg/mL (normal: < 3.0 pg/ML) and TNF-α 6.71 pg/mL (normal: < 3.10 pg/ML), but IL-4 0.1 pg/mL (normal: < 3.0 pg/ML) and IL-10 3.37 pg/mL (normal: < 4.1 pg/ML) were in normal range. The light microscopy showed isolated dyskeratotic cells and spicules with hyperkeratosis, and focal hyperkeratosis suggestive of DD (Fig. 1B). In addition, skin immunohistochemistry showed weak positivity for IL-6 (Fig. 1C). Mutation (c.193T>G:p.L65V) in the ATP2A2 gene was identified using whole-exome sequencing. Finally, she was diagnosed with DD. Isotretinoin led to a slight improvement, but even at small doses, there were intolerable side effects. She was distressed by itching and desired more treatment. After signing the informed consent form, she received treatment with Tocilizumab (8 mg/kg, repeated at 2-week intervals). During that time, she used emollients to supplement treatment. After 2-months of follow-up, forearm lesions (Fig. 2 A‒D) and itching were reduced, and sleep quality improved significantly.

**Figure 1:**
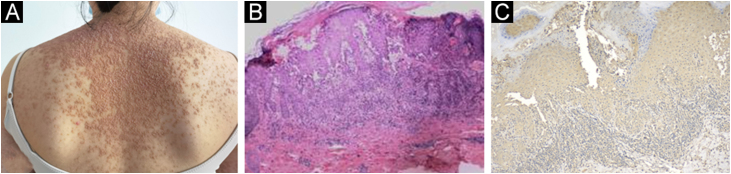
(A) Confluent red miliary papules on the back. (B) The light microscopy showed isolated dyskeratotic cells and spicules with hyperkeratosis and focal hyperkeratosis suggesting Darier disease. (C) Immunostaining with anti‐IL-6 (A11114, 1:200, ABclonal Biotech) showed weakly positive staining (x100).

**Figure 2:**
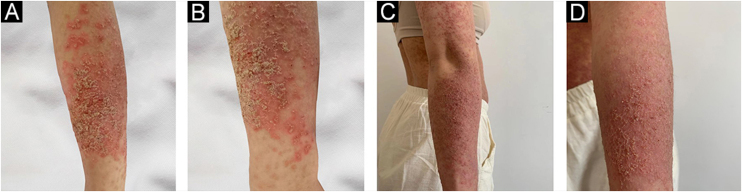
(A‒B) Multiple papules on her forearm. (C‒D) After the treatment marked improvement.

In in vitro experiments, UV light decreased the expression of the ATP2A2 gene, which is consistent with DD exacerbated by heat and UV light, and the gene expression of ATP2A2 was increased after the use of an IL-6 antibody.^2^ IL-6 antibodies have been proposed in the literature for the treatment of DD patients with summer exacerbations.^3^ Nevertheless, no cases of clinical use have been reported. Here, we found that Tocilizumab alleviates pruritus in summer exacerbations in DD patients and controls the further progression of skin inflammation. Biologicals can improve genodermatoses acting in the secondary inflammation and not in the mutation per se.

## Financial support

This work was supported by the Zhejiang Provincial Natural Science Foundation of China (LY22H160030).

## Authors' contributions

Lele Chen: Writing of the manuscript or critical review of important intellectual content.

Wenwen Wang: Data collection, or analysis and interpretation of data.

Sen Zhou: Statistical analysis.

Zhiming Li: Final approval of the final version of the manuscript.

## Conflicts of interest

None declared.

## References

[1] Haber R.N., Dib N.G. (2021). Management of Darier disease: a review of the literature and update. Indian J Dermatol Venereol Leprol..

[2] Mayuzumi N., Ikeda S., Kawada H., Ogawa H. (2005). Effects of drugs and anticytokine antibodies on expression of ATP2A2 and ATP2C1 in cultured normal human keratinocytes. Br J Dermatol..

[3] Takagi A., Kamijo M., Ikeda S. (2016). Darier disease. J Dermatol..

